# Symptom characteristics in patients undergoing acute type A aortic dissection surgery post-discharge phase: a prospective observational study

**DOI:** 10.1186/s40001-025-02495-6

**Published:** 2025-05-25

**Authors:** Jianlong Lin, Sailan Li, Yanchun Peng, Yaqin Chen, Liangwan Chen, Yanjuan Lin

**Affiliations:** 1 Department of Cardiovascular Surgery, Union Hospital, Fujian Medical University, No. 6, Xuefu South Road, Shangjie Town, Minhou County, Fuzhou, 350108, China; 2 Department of Nursing, Union Hospital, Fujian Medical University, No. 29, Xinquan Road, Fuzhou, 350001 China; 3 The School of Nursing, Fujian Medical University, No. 1, Xuefu North Road, Fuzhou, Fujian China

**Keywords:** Acute type A aortic dissection (AAAD), Symptom characteristics, Cardiac rehabilitation, Risk factors

## Abstract

**Objectives:**

In recent years, most studies on symptom characteristics in patients undergoing cardiac surgery have focused on the preoperative and postoperative phases. Relatively little knowledge is available related to the post-discharge phase. In this context, this paper aimed to analyze the symptoms and needs of patients with acute type A aortic dissection (AAAD) during the post-discharge phase.

**Methods:**

We recruited and studied patients who underwent acute type A aortic dissection surgery at Fujian Heart Medical Center from June 2022 to August 2023. At 3 months following the surgery, these subjects were investigated using the general information questionnaire and relevant symptom assessment scales, including the Mini-Mental State Examination Scale (MMSE), Athens Insomnia Scale (AIS), Hospital Anxiety and Depression Scale (HADS), and Fatigue Severity Scale (FSS). Meanwhile, grip strength and average step per day were measured for the exercise endurance assessment. A latent class analysis (LCA) based on the symptoms was performed, and differences in demographic and disease characteristics among different subgroups of patients were identified and compared using multivariate logistic regression.

**Results:**

A total of 228 patients were enrolled and categorized into three latent classes: fatigue–sleep disturbance (44.3%), anxiety–locomotion decline (16.9%), and high symptom groups (38.8%). Results showed that patients with cardiopulmonary bypass time > 200 min, higher BMI, or decreased grip strength were more likely to be classified as the high symptom group and those were unemployment status have a higher possibility of being defined as the anxiety–locomotion decline group.

**Conclusions:**

The symptom characteristics in patients with AAAD during the postoperative rehabilitation phrase exhibit heterogeneity. It is suggested that Clinical healthcare personnel improve the identification of symptoms in high-risk patients, particularly patients cardiopulmonary bypass time > 200 min, overweight or obese, unemployed status or decreased grip strength, relevant nursing interventions should be carried out to prevent the occurrence of surgical stress and complications in patients with AAAD early to improve the quality of life of patients.

## Introduction

Acute aortic dissection (AAD) is one of the most intricate and catastrophic cardiovascular conditions. Recently, the incidence of AAD has been increasing significantly every year [1]. Dissections that involve the arterial root, ascending aorta, or aortic arch are classified into Stanford type A or B. Approximately two-thirds of AAD cases are type A aortic dissections (AAADs), characterized by its sudden onset and rapid progression. AAD patients often experience co-occurring physical and psychological effects, which have a direct association with quality of life (QoL) [2]. They usually suffer from a variety of physical symptoms, such as drop in energy levels, fatigue, muscular aches and pains. Anxiety, psychological distress, and irritability are also commonly experienced [3]. The study demonstrated that patients undergoing cardiac surgery frequently experience preoperative weakness due to poor physical health, advanced age, prolonged sedation, unhealthy lifestyles, and anxiety. Simultaneously, extensive incisions, prolonged operation times, and lengthy general anesthesia reduce ventilation and physical activity, cause gastrointestinal dysfunction, and disrupt sleep–wake cycles, ultimately leading to a higher frequency of postoperative complications [4]. Neurological dysfunctions, particularly delirium [5] might be caused due to long-term mechanical ventilation, severe pain, and inflammatory responses in the surgical wounds. A decline in muscle strength may occur and last for several weeks or even months. According to the guidelines on cardiac rehabilitation by the Scottish Intercollegiate Guidelines Network, it is recommended to introduce more strategies for enhancing patients' self-efficacy in cardiac rehabilitation programs and monitoring symptoms of anxiety and depression [6]. With the increasingly clearer relationships between home-based cardiac rehabilitation and symptom progression following cardiac surgery, interventions designed for symptom characteristics in patients undergoing such surgery with a longer follow-up period have emerged as a burgeoning area of research. However, the lifestyle, dietary habits, work characteristics and cardiac rehabilitation completion during the post-discharge phase were often overlooked. The preoperative and postoperative phases of patients who have had cardiac surgery are mostly explored in current research on the latent classes of symptom characteristics.

## Patients and methods

### Study design

This is an observational prospective study. Patients with AAAD at Fujian Heart Medical Center between June 2022 and August 2023 were recruited. Inclusion criteria were: (1) the presence of AAAD, as suggested by computed tomography and MRI [8]; (2) postoperative stage of patients with aortic dissection; and (3) 18 years of age or older. The exclusion criteria were: (1) patients with trauma-induced AAAD and pregnant women; (2) complicated with heart failure, liver insufficiency and other important organ damage diseases; (3) patents ICU stay < 24 h; (4) history of readmission; or (5) patients without full medical records.

All the patients were admitted to the intensive care unit and were given sedation, analgesia, oxygen inhalation, and keep a normal bowel movement. Urapidil and sodium nitroprusside were used to control the systolic blood pressure within 100–120 mmHg (1 mmHg = 0.133 kPa), and β-receptor blockers were used to control the heart rate within 60–80/min. We researched AAAD patients with cear diagnosis by CTA and almost all of them have undergone implantation of modified triple-branched stent graft for descending aorta replacement in addition to aortic root reconstruction and ascending aorta or hemiarch replacement. The rest in Stanford type B were treated with thoracic endovascular aortic repair (TEVAR).

All patients performed heart disease rehabilitation based on non-invasive cardiac output measurement system at hospital. For patients after discharge from hospital, early identification of disability patients and cardiac rehabilitation comprehensive strategies such as reasonable blood lipid management, drug prevention, healthy lifestyle adjustment, and exercise intervention can achieve the goals of controlling the occurrence and development of disability after discharge from hospital.

### Data collection

Sociodemographic and clinical data of the patients were collected by checking the electronic medical records. Sociodemographic data including age, sex, body mass index (BMI), marital status, educational background, preoperative mini-mental state examination (MMSE) [9], and anxiety and depression levels were obtained. The clinical data of patients such as hypertension, diabetes, cardiopulmonary bypass (CBP), aortic clamping time and extension of aortic dissection were documented. The primary outcome indicators for complications were postoperative pulmonary complications, and ICU length of stay. Other data including the lifestyle (insufficient physical activity and irregular sleep schedule) following hospital discharge, and occupation were collected. In addition, nutritional status was assessed using NRS2002 (Nutritional Risk Screening 2002) Nutritional score. A nutritional risk screening NRS2002 score of 3 indicates that patients have a nutritional risk [10]. Information related to cognitive and physical functions as well as mental health after surgery was included in the study. Use the following universality scale evaluates whether the patient has cognitive, psychological and physiological aspects disorder, where the assessment of any of the scales is positive is diagnosed. Cognitive function was assessed using MMSE from domains of orientation, repetition, attention and calculation, verbal recall, and language. The "*Z*-score" method was employed in this study, where *Z* = (change value compared to the initial assessment–mean change value)/standard deviation of change value. When there are 2 or more of the 5 above-mentioned assessments with *Z* values ≥ 1.96, cognitive decline is diagnosed. As for the mental health, assessments related to sleep and psychological status were conducted. The sleep status was assessed using the Athens insomnia scale (AIS) from 8 aspects, including the onset time of sleeping, early awakening, nocturnal awakenings, sleep quality, sleep duration, daytime sleepiness, daytime physical function, and daytime emotional state [11]. Patients' anxiety and depression levels were evaluated using the hospital anxiety and depression scale (HADS) [12]. Grip strength assessments were conducted by responsible nurses using a dynamometer before discharge and at 3 months postoperatively. A dynamometer (CAMRY® Electronic Hand Dynamometer, China) was used, and maximum force was applied at a constant speed, with visual feedback and verbal encouragement avoided during the process. Grip strength was measured twice for each hand, with an interval of more than 15 s to avoid muscle fatigue. Postoperative physical function assessed by the six-minute walk test (6 MWT) for exercise capacity. Fatigue Severity Scale (FSS) was utilized to evaluate fatigue severity [13].

The main outcome indicators of poor prognosis were adverse outcomes including new arrhythmias, cardiac insufficiency, acute pericardial tamponade, disturbance of consciousness, dyskinesia, intracerebral hemorrhage or hematoma, cerebral infarction, transient ischemic attack, and central nervous system infection. Secondary outcome measures included postoperative acute kidney injury, postoperative pulmonary complications, prolonged intensive care unit (ICU) mechanical ventilation, stay time, and postoperative hospital stay. Prolonged mechanical ventilation (PMV) was defined as a duration of mechanical ventilation exceeding 48 h. Postoperative AKI was defined as an increase in serum creatinine (Scr) ≥ 0.3 mg/dl within 48 h or Scr ≥ 1.5 times the baseline level within 7 days. Demographic data of patients were collected based on the electronic medical records and other necessary data absent in the medical records were obtained through inquiries with patients or their family members. At 3 months after surgery, researchers conducted follow-up surveys on rehabilitation-related symptoms through questionnaires.

### Statistical analysis

A statistical method of Latent class analysis (LCA) was used to describe and distinguish research subjects into various subgroups based on their significant features [7]. We introduced a model to analyze the latent classes. The number of the categories of the model individually was enlarged and the fitting indicators among models were compared. *P* values below 0.05 indicate that the *k* class model is selected over the *k* − 1 class model. The individual class was determined based on posterior probability, and the number of latent classes of symptom characteristics was explored. Each latent class was named in light of the changing trends and characteristics of the class. Comparisons of baseline characteristics between classes were carried out. The measurement data accorded with normal distribution were expressed as mean ± standard deviation. In addition, we analyzed the continuous variables through analysis of variance and tested the categorical variables using the chi-square test. The chi-square test was introduced to count data expressed as percentages. The measurement data of non-normal distribution were compared using the Kruskal–Wallis test and are expressed as median (interquartile interval). The chi-square test was used for counting data which are expressed as percentages. Meanwhile, multiple logistic regression analysis was conducted to determine the risk factors, in which the impact of social demography and disease-related data on symptom characteristics was explored. The nomogram gives the total score by adding the corresponding scores of each related factors and gives the probability of the point on the risk axis with the total score. We also evaluated the correlation between different factors of latent classes by calculating the odds ratios (ORs) and 95% confidence intervals (CIs). Based on these independent risk factors, a nomogram model was developed to predict the risk of high symptom of patients. Moreover, the area under the curve (AUC) of receiver operating characteristic curve (ROC) was used to evaluate the model differentiation, the Hosmer–Lemeshow goodness-of-fit test (H–L test) and calibration curve were used to evaluate the calibration degree of the model. SPSS software (version 22.0) and Mplus8.4 were used for data analyses. In addition, piecewise linear regression was used to quantify the odds ratio (OR) per 1 kg grip strength increase below and above the change point. Statistical significance was considered as two-sided *p* < 0.05.

## Results

### Patient clinical characteristics

**Fig. 1:**
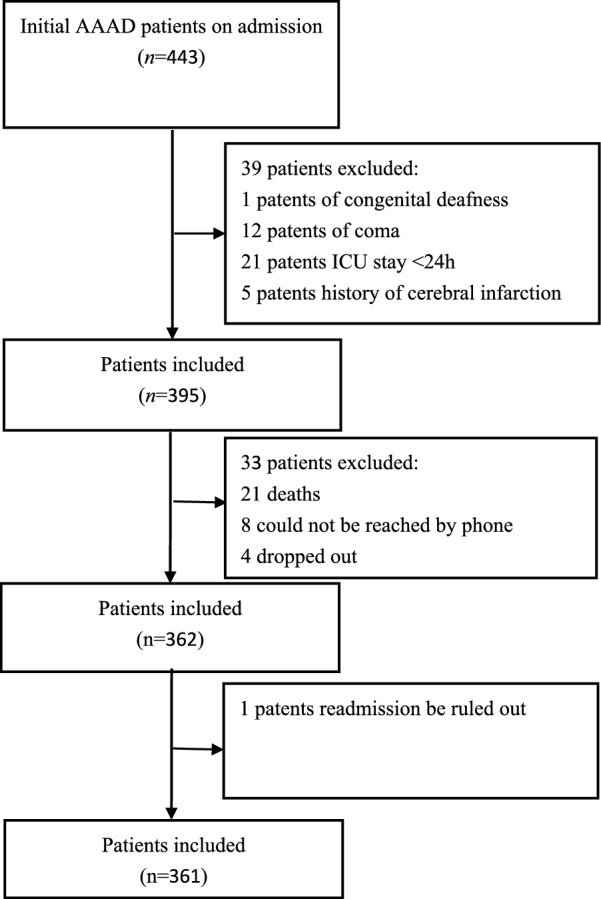
Study flow chart

361 of 443 patients with AAAD were included in this study (as shown in Fig. 1) with a mean age of 52.54 ± 11.25 years (288 male; 73 female). Poor prognosis was reported in 80 subjects. Pulmonary complications was documented in 15 subjects (Table 2).

### Distribution of symptom characteristics in patients with AAAD

Among 361 patients with AAAD, declining cognition, psychology, and physiology functions were present in 228 cases (63.16%), the characteristics of the cognitive, psychological and physical impairments including 96 of cognitive dysfunctions (41.67%), and 147 of sleep disorders (64.47%). In addition, 56 subjects suffered fatigue (24.56%), 91 less physical activity (39.91%), 161 anxiety (71.05%), and 64 depression (28.07%).

### Identification and determination of latent classes of symptom

The symptom characteristics including cognitive dysfunctions, anxiety, depression, decreased physical activity, sleep disorders, and fatigue were used as indicators in the observation. The LCA was used for a free estimation of the time parameters, and 1–4 categories were extracted. When the number of potential category number increased from 1 to 3, AIC, BIC, and aBIC decreased, and BLRT and VLMR reached significant levels (*P* < 0.001). AIC, aBIC, and BIC values increased from three to four categories, and BLRT reached a significant level in five categories (*P* > 0.05). Finally, three subgroups of the LCA were retained (Table 1).

**Table 1:** Fit indices of latent class analysis on symptom characteristics

No. of	LL	AIC	BIC	a-BIC	Entropy	BLRT(P)	VLMR(P)	N
1	− 415.516	843.032	854.966	836.116	–	–	–	–
2	− 394.494	806.987	824.888	796.613	0.998	*P* < 0.001	0.0015	74.1/25.9
3	− 373.928	771.856	795.724	758.023	0.982	*P* < 0.001	0.0053	44.3/16.9/38.8
4	− 364.479	758.959	788.794	741.668	0.962	*P* < 0.001	0.2840	31.5/24.1/25.9/18.5

LL, log likelihood; AIC, akaike information criterion; BIC, Bayesian information criterion; a-BIC, adjusted Bayesian information criterion; BLRT, bootstrapped likelihood ratio test; VLMR, Vuong Lo-Mendell-Rubin likelihood ratio test

**Fig. 2:**
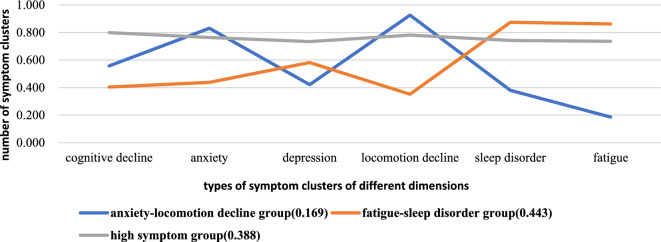
Distribution of three latent classes of symptom characteristics

In Group l (orange), a higher prevalence of fatigue and sleep disorders and a lower prevalence of cognitive decline, anxiety, and decreased physical activity were reported, which was classified as a "fatigue-sleep disorder group". While the number of patients with cognitive decline and depression was moderate in group 2 (blue), patients in this group had a higher incidence of anxiety and decreased physical activity, and sleep disorders and fatigue were less observed. Therefore, group 2 was considered an "anxiety–locomotion decline group". Patients in group 3 were found to have a higher occurrence of all symptoms (grey), making it a "high symptom group" (Fig. 2).

### Univariate and multivariate analyses

The univariate analysis documented that age and CPB time in the anxiety–locomotion decline group were lower and unemployed status was higher than those in the other groups (*P* < 0.05). There were significant differences between LVEF < 50 (7.90 vs. 5.10 vs. 19.30%) and pulmonary complications (5.90 vs. 15.40 vs. 3.40%). Grip strength were different in the three groups (*P* < 0.05). 24 patients (61.50%) unemployed in the anxiety–locomotion decline group, 38 patients (37.60%) in the fatigue–sleep disorder group, and 39 patients (44.30%) in the high symptom group. Meanwhile, it was recorded that the three groups differed in their cardiac rehabilitation completion (*P* < 0.05) (Table 2).

**Table 2:** Baseline characteristics in three latent classes

	fatigue–sleep disorder group (*n* = 101)	anxiety–locomotion decline group (*n* = 39)	high symptom group (*n* = 88)	*P*
Sociodemographic characteristics				
Age (y), mean (SD)	52.88 ± 11.40	47.59 ± 10.42	52.49 ± 8.01	**0.036**
Male, *n* (%)	78 (77.20)	31 (79.50)	75 (80.95)	0.372
BMI (kg/m^2^), mean (SD)	24.6 ± 3.4	24.6 ± 3.3	26.1 ± 3.3	**0.033**
Extension of AAAD, *n* (%)				0.469
Ascending aorta	16 (15.80)	7 (17.90)	20 (22.70)	-
Aortic arch	2 (2.00)	1 (2.60)	1 (1.10)	-
Descending aorta	28 (27.70)	5 (12.80)	17 (19.30)	-
Abdominal aorta and/or beyond	55 (54.50)	26 (66.70)	50 (56.80)	-
Preoperative				
Hypertension, *n* (%)	98 (98.00)	38 (97.40)	86 (97.70)	0.978
Diabetes mellitus, *n* (%)	3 (3.00)	3 (7.70)	4 (4.50)	0.471
MMSE score, mean (SD)	27.96 ± 3.85	28.33 ± 1.72	27.81 ± 1.90	0.339
HADS score, mean (SD)				
Anxiety	7.18 ± 3.85	7.19 ± 2.89	7.07 ± 3.39	0.597
Depression	8.94 ± 1.35	8.97 ± 1.27	9.15 ± 1.62	0.972
Intraoperative				
Operating time (min), mean (SD)	312.70 ± 62.40	298.14 ± 58.94	305.89 ± 66.16	0.474
CPB time > 200 min, *n* (%)	69 (68.30)	26 (66.70)	73 (83.00)	**0.041**
Aortic clamping time (min), mean (SD)	82.20 ± 42.84	81.95 ± 4149	83.92 ± 51.75	0.051
Perfusion temperature				
Deep hypothermia	10 (9.90)	4 (10.26)	14 (15.91)	0.556
Surgical management				
Root replacement, *n* (%)	3 (3.00)	3 (7.70)	4 (4.50)	0.471
Additional CABG, *n* (%)	0 (0.00)	0 (0.00)	2 (2.30)	0.201^b^
Postoperative				
LVEDD, mm, median (IQR)	38(36,53)	43(39,51)	43(37,51)	0.958^a^
LVEF < 50, *n* (%)	8 (7.90)	2 (5.10)	17 (19.30)	**0.019**
Post-discharge				
Grip strength, *n* (%)	26.90 ± 2.98	26.62 ± 3.67	25.78 ± 2.74	**0.044**
Six-minute walk test < 450 *m*, *n* (%)	12 (11.88)	5 (12.82)	13 (14.77)	0.556
Malnutrition, *n* (%)	0 (0.00)	0 (0.00)	2 (2.30)	0.201^b^
Unhealthy lifestyle behaviors				
Irregular sleep schedule, *n* (%)	54 (53.50)	23 (59.00)	56 (63.60)	0.366
Occupation, *n* (%)				
Unemployed, *n* (%)	38 (37.60)	24 (61.50)	39 (44.30)	**0.038**
Cardiac rehabilitation completion, *n* (%)	57 (56.40)	18 (46.20)	32(36.40)	**0.022**
Outcomes				
Length of ICU stays (d), median (d)	8.81 ± 8.71	9.73 ± 10.63	11.03 ± 11.56	0.331
Hospital stays(day), median (IQR)	21 (16, 30)	23 (17, 34)	23 (16, 32)	0.077^a^
In-hospital Complications				
Pulmonary complications, *n* (%)	6 (5.90)	6 (15.40)	3 (3.40)	**0.040**
Acute kidney injury, *n* (%)	11 (10.89)	5 (12.82)	21 (23.86)	**0.043**
Prolonged MV, *n* (%)	9 (8.91)	5 (12.82)	23 (26.13)	**0.046**
Arrhythmia, *n* (%)	5 (4.95)	1 (2.56)	11 (12.50)	**0.006**
Poor prognosis, *n* (%)	27 (26.73)	10 (25.64)	43 (48.86)	**0.005**

BMI, body mass index; LVEDD, left ventricular end-diastolic diameter; LEVF, left ventricular ejection fraction; HADS, hospital anxiety and depression scale; CPB, cardiopulmonary bypass; ICU, intensive care unit; MV, mechanical ventilation; SD, standard deviation; IQR, interquartile range

^a^Kruskal–Wallis *H*

^b^Fisher's exact test

**Fig. 3:**
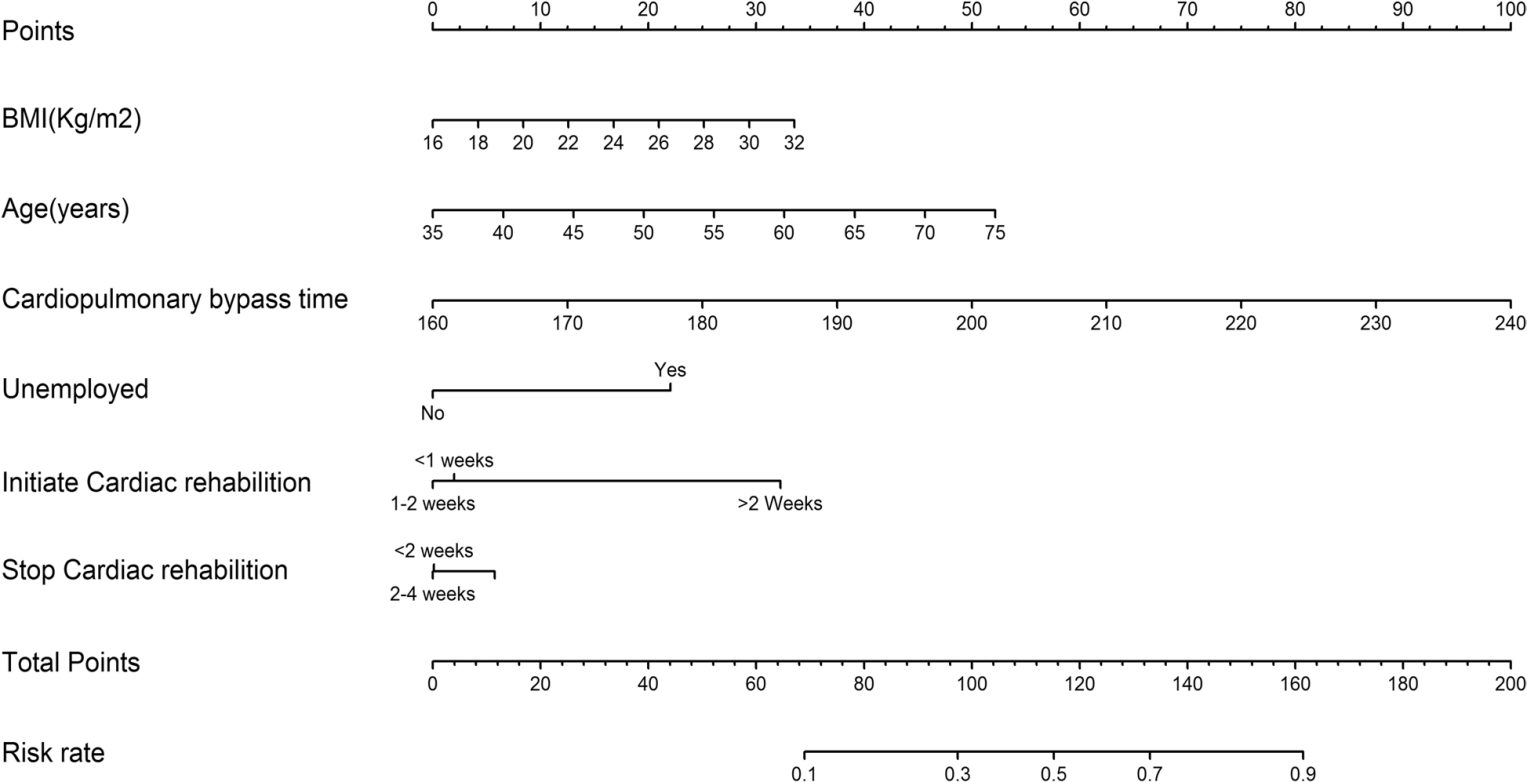
Nomogram to estimate risk of high symptom of patients with acute type A aortic dissection

Based on the results of the univariate analysis and collinear diagnosis, variables with *P* < 0.05 were included to screen for independent risk factors in the multiple regression analysis. Age, CPB time, grip strength, and unemployed status were independent risk factors of 3 latent classes. According to the analysis, the probability of different influencing factors being attributed to different groups are shown in Table 3. Compared with the fatigue–sleep disorder group, higher BMI (OR = 0.965, 95% CI 0.939–0.993, *P* = 0.014), grip strength (OR = 1.418, 95% CI 2.103–8.108, *P* = 0.000), and CPB time > 200 min (OR = 1.163, 95% CI 1.455–7.031, *P* = 0.004) showed a higher possibility of being classified into the high symptom group. Compared with the fatigue–sleep disorder group, unemployed status patients (OR = 3.087, 95% CI 1.238–7.697, *P* = 0.016) were more likely to be included into the anxiety–locomotion decline group. The logistic regression analysis showed that BMI (OR = 1.073,95%CI 1.027–1.122, *P* = 0.002), age (OR = 1.046,95%CI 1.016–1.077, *P* = 0.003), unemployment status (OR = 2.064,95%CI 1.010–4.219, *P* = 0.047), cardiac rehabilitation duration (OR = 1.529,95%CI 1.058–2.208, *P* = 0.024) and CPB time (OR = 1.833,95%CI 1.574–2.134, *P* < 0.001) were independent risk factors for high symptom of patients with AAAD. The nomogram was plotted according to independent risk factors (Fig. 3).

**Table 3:** Multiple logistic regression of 3 latent classes

Variances	B	SE	Wald value	*P*	OR	95%CI
C3 vs. C1						
CPB time > 200 min	0.921	0.379	5.908	0.015	2.512	1.195–5.281
BMI > 28 kg/m^2^	−0.981	0.429	5.221	0.022	0.375	0.162–0.870
Grip strength	1.072	0.480	4.989	0.026	2.922	1.140–7.488
C3 vs. C2						
CPB time > 200 min	1.080	0.478	5.098	0.024	2.945	1.153–7.523
BMI > 28 kg/m^2^	−2.245	1.064	4.451	0.035	0.106	0.013–0.853
C1 vs. C2						
Unemployed status	0.928	0.460	4.071	0.044	2.529	1.027–6.229

C3 (high symptom group), C2 (anxiety–locomotion decline group), C1 (fatigue–sleep disorder group)

CPB, cardiopulmonary bypass; OR, odds ratio; CI, confidence interval

### Distinguish ability of the model

**Fig. 4:**
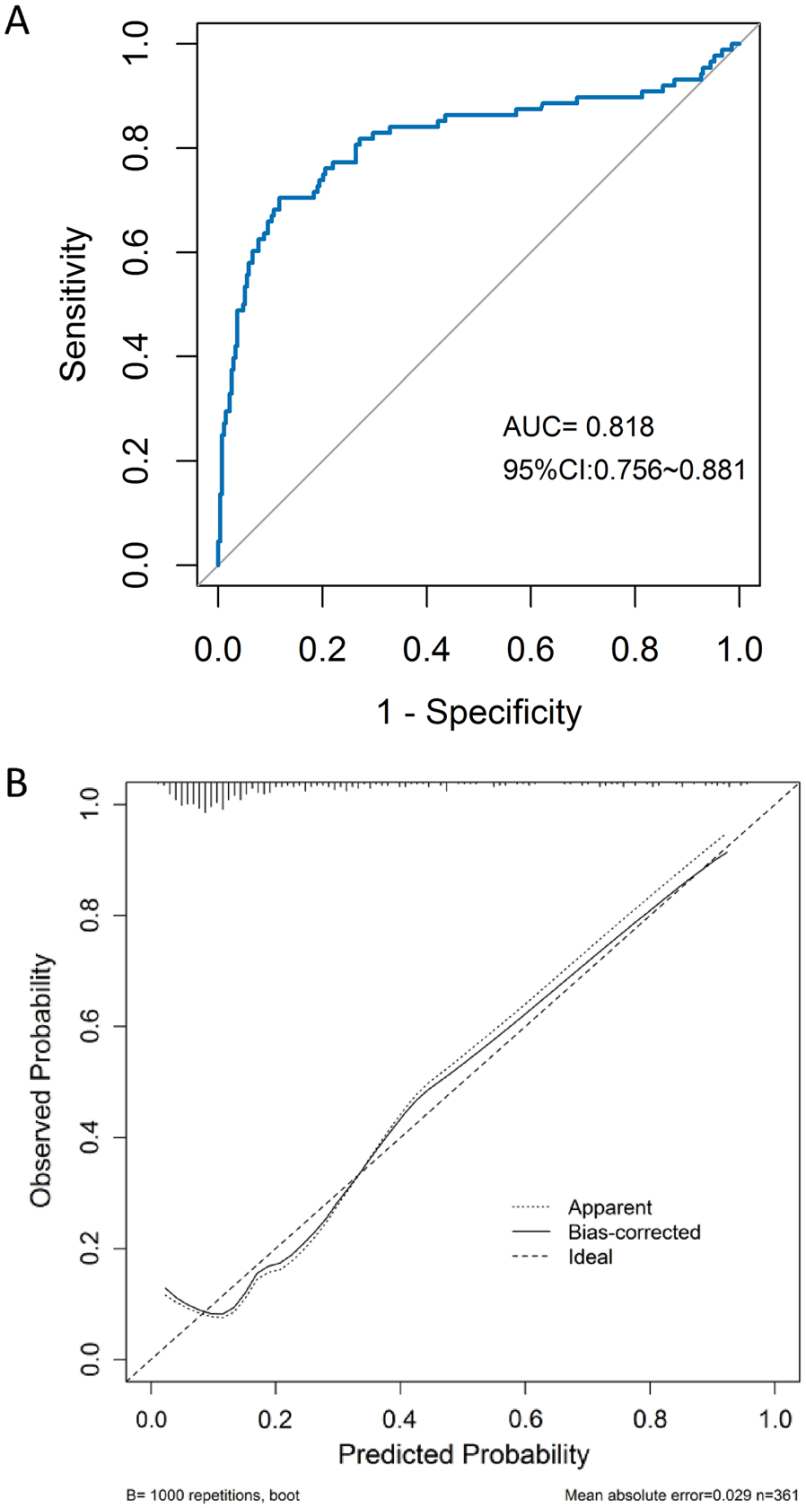
**A** Calibration curve of nomogram mode. **B** Receiver operator characteristic curve

ROC curves examine the predictive ability of each of the factors obtained, and select the factor with the highest predictive ability, and the results showed an AUC of 0.818 (95% CI 0.756–0.881, *P* < 0.001), a sensitivity of 77.5% and a specificity of 88%, suggesting that the model has a good discrimination. The ROC curve of prediction model is shown in Fig. 4A.

### Calibration degree of the model

The results of the H–L test showed *χ*^2^ = 8.709, *P* = 0.367 > 0.05, and the calibration curve was well fitted as shown by bootstrap method with 1000 repetitions (Fig. 4B), indicating that our predicted and observed values are close and in good agreement.

**Fig. 5:**
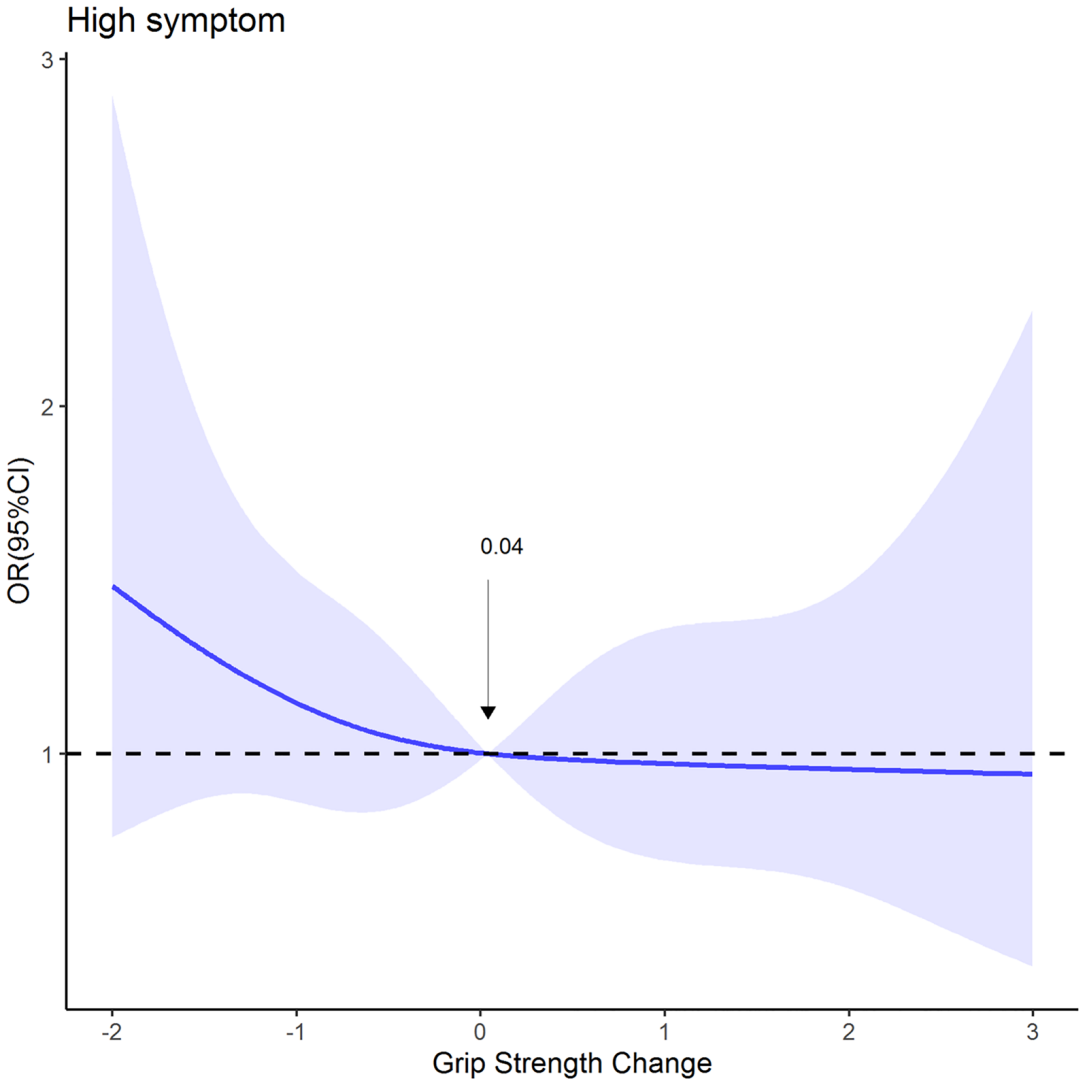
Association between grip strength change and high symptom based on the restricted cubic spline model

The piecewise linear regression analysis showed that (Fig. 5), the estimated OR of the grip strength per 1 kg increase in high symptom group was 0.638 (95% CI 0.422–0.965) below 0.04 µmol/L and 0.974 (95% CI 0.714–1.328) above this point.

## Discussion

Some studies have shown the high prevalence of disorders in patients with CPB. However, does not delve into the underlying mechanisms, requiring further research into the biological and psychological processes. In this context, LCA was used to categorize symptom subgroups, aiding in understanding varied post-discharge experiences and the need for personalized post-discharge care. As far as we know, this study pioneers in exploring the incidence of cognitive, physical, and psychological symptoms in patients with AAAD throughout the post-discharge phase and analyzing the latent classes of symptom characteristics and risk factors. The results showed that: (1) 63.31% of patients undergoing the AAAD surgery had various degrees of cognitive, psychological, and physiological dysfunctions during the postoperative discharge phase. (2) 38.8% of the 228 subjects were in the "high symptom group", 16.9% in the "anxiety–locomotion decline group", and 44.3% in the "fatigue–sleep disorder group". (3) Patients with decreased grip strength, overweight or obesity, and CPB time > 200 min had a higher risk of being included in the high symptom group. Furthermore, the anxiety–locomotion decline of patients who were currently unemployed was more significant than that of the other subgroups.

With the latent class model, three potential subgroups were obtained: the high symptom group, the anxiety–locomotion decline group, and the fatigue–sleep disorder group. The proportion of patients in the fatigue–sleep disorder group was the highest (44.0%). Most patients with AAAD have myocardial hypertrophy, and the aortic valve is frequently involved in dissection, resulting in inadequate myocardial contractility during cardiac resynchronization [14]. This study showed that patients in the fatigue–sleep disorder group are more likely to have a low LVEF (7.90%), which is due to that changes in sleep structure can cause sleep disorders. Due to special pathophysiological changes such as stimulation of the vagus nerve in the lung, patients with lower LVEF were more susceptible to central sleep apnea. Which may led to frequent awakening during sleep and disrupted the maintenance of slow wave sleep. Besides, prolonged bed rest during daytime may lead to the reduction of slow wave sleep at night, thus changing sleep structure [15]. Current studies have shown that sleep disorders in patients undergoing cardiac surgery might be associated with advanced age, pain, atrial fibrillation, inflammatory factors, drug use, and cognitive dysfunction [16]. Therefore, clinicians should actively ascertain the causes of persistently sleep disorder in the early stages of patient care and promptly take targeted measures to prevent clinical complications.

This study also found that the anxiety–locomotion decline group accounts for 16.9% of patients. Particularly, the anxiety–locomotion decline of patients with unemployment status was more significant than that of the other subgroups. This result is consistent with that of Yan et al. [17], which found that unemployed and retired patients have higher levels of locomotion decline. Studies indicate that individuals with higher occupational self-awareness are less likely to develop exercise decline. Many people strongly desire to return to their previous jobs, which are closely tied to their lifestyle and performance expectations. Occupational self-awareness influences career choices and behavior patterns. When individuals engage in valued professions, they actively participate in rehabilitation to return to work sooner. This suggests healthcare providers should recognize the positive impact of occupational roles and self-awareness on rehabilitation. Supporting patients' occupational identities and goals in treatment plans can enhance motivation, speed up recovery, and improve overall satisfaction. In fact, other deeper work motivation-related mechanisms still need to be further confirmed by large sample surveys or intervention studies in the future.

The results of this study indicated that the high symptom group accounting for 38.8% of patients was recorded. The logistic regression to extract independent predictors of high symptom. This analysis showed that BMI, age, unemployment status, decreased grip strength, cardiac rehabilitation duration and CPB time were independent risk factors for high symptom of patients with AAAD. Compared with the other groups, the multivariate regression analysis in this study showed that patients with decreased grip strength, overweight or obese, and CPB time > 200 min registered a higher risk of being included in the high symptom group. This result is consistent with that of Li et al. [18], who studied patients with acute thoracic aortic dissection who were obese. Particularly, patients who were morbidly obese had a high risk ratio of postoperative pulmonary complications and poor prognosis. Simultaneously, compared to patients with a normal BMI, those with higher BMI have relatively higher energy expenditure or requirements. Conversely, a meta-analysis showed no difference in adverse outcomes between patients of normal weight and patients who were overweight or obese. Although the difference was not significant, the poor prognosis of patients with abdominal obesity was higher than that of other patients, suggesting that different types of obesity may affect patient prognosis. This concept requires further clinical confirmation. Aortic dissection often requires cardiopulmonary CPB, increasing the probability of serious complications such as those affecting the nervous or renal systems. In addition, Patients such as those in this study who underwent CPB are prone to aggravate the body burden resulting from surgical trauma and long duration of stress stimulation. Grip strength can be used to assess a patient's muscle strength, which, along with functional independence, is a key determinant of frailty. Prolonged decline in muscle strength and restricted activity increase the likelihood of frailty and pre-frailty. Therefore, healthcare professionals should focus on preventing and treating decreased grip strength.

It is undeniable that this study has some limitations. Efforts should be made to determine if cultural factors play a more significant role than evident in this study. Data was not captured that indicated whether the patient or family made the decision to discontinue cardiac rehabilitation. Future studies should define additional data points for study, and consider incorporating additional information requests about non-clinical reasons for better outcomes, and think about using whether develop disability after discharge as a component to add other predictive ratios, its application in AAAD and related research on prognosis prediction. Meanwhile, we did not include physical function status at hospital discharge data in our analysis. In the later research, we will consider evaluating the disability at hospital discharge can provide patients with a basis for rehabilitation programs and symptomatic intervention.

## Conclusion

The symptom characteristics during the postoperative rehabilitation period in patients with AAAD exhibit heterogeneity, CPB time > 200 min, overweight or obese, unemployed status or decreased grip strength can increase the risk of different symptom classes of patients with AAAD after surgery. Further studies are required to formulate targeted interventions for different classes of patients, thus enabling faster postoperative recovery.

## Data Availability

The data that support the findings of this study are available from the corresponding author on reasonable request. No datasets were generated or analysed during the current study.
